# Twins discordant for myositis and systemic lupus erythematosus show markedly enriched autoantibodies in the affected twin supporting environmental influences in pathogenesis

**DOI:** 10.1186/1471-2474-15-67

**Published:** 2014-03-06

**Authors:** Lu Gan, Terrance P O’Hanlon, Aaron S Gordon, Lisa G Rider, Frederick W Miller, Peter D Burbelo

**Affiliations:** 1 Environmental Autoimmunity Group, National Institute of Environmental Health Sciences, Bethesda, MD, USA; 2 Dental Clinical Research Core, National Institute of Dental and Craniofacial Research, National Institutes of Health, Bethesda, MD, USA

## Abstract

**Background:**

Studies of twin pairs discordant for autoimmune conditions provide a unique opportunity to explore contributing factors triggered by complex gene-environment interactions.

**Methods:**

In this cross-sectional study, thirty-one monozygotic or dizygotic twin pairs discordant for myositis or systemic lupus erythematosus (SLE), along with matched healthy controls were evaluated for antibodies against a panel of 21 autoantigens.

**Results:**

Autoantibody profiling revealed that 42% of the affected twins showed significant seropositivity against autoantigens in the panel. In many of these affected twins, but none of healthy controls, there were high levels of autoantibodies detected against two or more autoantigens commonly seen in systemic autoimmune diseases including Ro52, Ro60, RNP-70 K and/or RNP-A. In contrast, only 10% (3/31) of the unaffected twins showed seropositivity and these immunoreactivities were against single autoantigens not seen in systemic autoimmune diseases. While no significant differences in autoantibodies were detected between the affected or unaffected twins against thyroid peroxidase, transglutaminase and several cytokines, 23% of the affected twins with myositis showed autoantibodies against the gastric ATPase. Analysis of the monozygotic twins separately also revealed a higher frequencies of autoantibodies in the affected twins compared to the unaffected twins (*P* = 0.046). Lastly, clinical analysis of both the affected monozygotic and dizygotic twins revealed that the autoantibody seropositive affected twins had a greater global disease activity score compared to seronegative affected twins (*P* = 0.019).

**Conclusion:**

The findings of significantly more autoantibodies in the affected twins with myositis and SLE compared to the unaffected twins are consistent with potential non-genetic factors playing a role in autoantibody production and pathogenesis of these autoimmune disorders.

## Background

Factors involved in the development of various autoimmune diseases including type I diabetes (T1D), rheumatoid arthritis, myositis, and systemic lupus erythematosus (SLE) remain poorly defined. Studies of monozygotic twins with autoimmune conditions including multiple sclerosis, T1D, rheumatoid arthritis and SLE show a concordance rate of 20-70%, suggesting that the genetic makeup alone cannot completely explain the pathogenesis of autoimmunity [1]. Consistent with these findings, genetic linkage studies have identified a limited number of susceptibility genes responsible for different autoimmune conditions, many of which play important roles in immune function [2]. Increasing evidence suggests that environmental factors, including epigenetic DNA methylation, chemical exposures, and host-pathogen interactions, may trigger autoimmune conditions in genetically susceptible individuals [3-5]. Specific environmental exposures, including pathogens, as well as with the timing of exposure or infection, are of particular interest because they may profoundly impact immune function and may promote autoimmunity [6].

Systemic autoimmune diseases including systemic sclerosis, rheumatoid arthritis, SLE and idiopathic inflammatory myopathies (IIM) or myositis, are a group of immune disorders characterized by immune activation, autoantibody production and tissue destruction involving multiple organs. Although the target tissues and symptoms are markedly different among these diseases, a common molecular feature is the increased expression of interferon-regulated genes [7]. For example, gene expression studies have shown that SLE, Sjögren’s syndrome and dermatomyositis (DM, a subtype of IIM with characteristic cutaneous findings) share up-regulated expression of type 1 interferon response genes [8-11]. In some cases, increased interferon-α has been reported in patients with systemic autoimmune disease, whereby this cytokine likely drives inflammation in target tissues [12-14].

Autoantibodies are another common feature of these diseases, which have important clinical and prognostic utility [15]. A number of autoantibodies such as anti-DNA, anti-Smith (Sm) and antinuclear antibodies (ANA) have been selected as classification criteria for SLE [16]. Some autoantibodies are myositis-specific and include those reactive against aminoacyl-tRNA synthetases, signal recognition particle, Ku, Mi-2, p155/140, and PM/Scl [17]. However, autoantibodies against other targets, such as SSA and SSB, are not disease-specific and can be detected in patients with most systemic autoimmune conditions. Besides assisting with clinical diagnosis, the levels of autoantibodies can correlate with specific symptoms and disease severity [15]. In some cases, autoantibodies have also been detected before clinical symptoms, thereby providing insight into the temporal onset of autoimmune disease [15]. In one seminal study in SLE, autoantibodies against several targets, including DNA, SSA, SSB and Sm antigens, were detected before the clinical onset of SLE [18].

However, despite the widespread applications of autoantibody testing, only a limited number of studies have utilized autoantibody profiling to study cohorts of twins to dissect genetic and environmental factors that may contribute to systemic autoimmune disorders. In one small study of seven twin pairs discordant for SLE, 50% of the affected twins were found to have anti-DNA antibodies [19]. Other studies have used first degree relatives of SLE patients instead of twins and have found higher ANA autoantibody levels in those unaffected, related subjects than those seen in unrelated, healthy controls [20,21].

Recently, we have employed a liquid phase immunoassay, luciferase immunoprecipitation systems (LIPS), which utilizes light-emitting recombinant antigens to efficiently detect antibodies against linear and conformational epitopes associated with a variety of human autoantigens and infectious agents [22]. Due to the wide dynamic range of detection and low backgrounds, LIPS has been highly informative in characterizing autoantibody levels in multiple autoimmune conditions, including patients with opportunistic infections [23], Sjögren’s syndrome [24], T1D [25], Stiffman syndrome [26], myasthenia gravis [27] and SLE [28]. In T1D, Sjögren’s syndrome and SLE, LIPS identified unique patient autoantibody profiles that potentially associated with disease subsets [24,25,28]. Here we describe our findings profiling antibodies against a panel of autoantigens in a cohort of 31 twin pairs discordant for myositis or SLE along with control subjects.

## Methods

### Ethics statement and study population

Informed written consent was obtained from all subjects in accordance with the human experimentation guidelines of the Department of Health and Human Services under multiple IRB-approved protocols, and the studies were conducted according to the principles expressed in the Declaration of Helsinki. Under IRB approved protocols at the National Institute of Environmental Health Sciences, NIH (Bethesda, MD), 31 same-gendered twin pairs discordant for two different systemic autoimmune conditions (22 myositis and 9 SLE) were studied as part of the Twin-Sib study [29,30]. From the 22 affected twins with myositis, 19 children had dermatomyositis (DM), two adults had DM and one had polymyositis (PM) contingent on the Bohan and Peter criteria [31,32]. Based on American College of Rheumatology criteria, there were five children and four adults with SLE. Both children and adults with SLE or IIM were enrolled into the study within four years of the diagnosis. Patients with inherited, metabolic, infectious, or other known causes of disease were excluded. In addition to the twin pairs, 31 unrelated healthy controls that were matched for age, gender and ethnicity, were enrolled from the NIH healthy control registry and underwent medical evaluation. These controls were free of trauma, infections, surgeries and vaccinations for at least eight weeks before enrollment and had no first-degree family members with systemic autoimmune diseases. The physician performed global assessments of disease activity and disease damage based on all available information, including the patient’s appearance, medical history, physical examination, laboratory test result, and any medical therapy. The scores of the affected twins were then recorded by a 0-100 mm visual analogue scale [29,33]. The scales from 0 to 100 indicate inactive disease/no damage to most severe disease activity/damage. The available information including age, gender, monozygotic/dizygotic status, and clinical information including specific diagnosis, treatment status and other clinical laboratory findings of the patients are summarized in Table 1.

**Table 1:** Clinical Characteristics of 33 Affected Twins

	**Discordant Affected Twins**
Diagnosis	24 dermatomyositis, 6 systemic lupus erythematosus and 1 polymyositis
Number of Monozygotic Twins	71% (22/31)
Number of Dizygotic Twins	29% (9/31)
Age (mean, ± SD)	14.2 + 2.1 years
Disease Duration (mean, ± SD)	1.69+ 1.5 years
Gender (% Female)	61% (19/31)
Taking Immunosuppressive Treatment^a^	93.5% (29/31)
Global Disease Activity Score^b^ (mean, ± SD)	20.2 + 17.3
Global Disease Damage Score^c^ (mean, ± SD)	7.8 + 15.8

^a^Treatment included prednisone and any immunosuppressive regimen.

^b^Global Disease Activity score was assessed by a physician familiar with the subject on a 0-100 mm visual analogue scale.

^c^Global Disease Damage score was assessed by a physician familiar with the subject on a 0-100 mm visual analogue scale.

### LIPS antigens

LIPS tests for many of the autoantigens used in this study have been previously described and included targets for systemic autoimmune diseases (Ro52, Ro60, La, Sm-D3 (Sm), Jo-1, RNP-A, and RNP-70 K), tissue autoantigens (thyroid peroxidase/TPO), transglutaminase/TGM2), the gastric ATPase and cytokines (interleukin-1α/IL-1 α, interferon-α/IFN-α, interferon-ω/ IFN-ω, interferon-γ/ IFN-γ, interleukin-17/IL-17, GMCSF, and TNF-β) [23,24,28]. Of note, the SSA LIPS test separately detects autoantibodies against Ro52 and Ro60 antigens and SSB is directed against the recombinant La protein. Several new *Renilla* luciferase fusion protein constructs were generated for detecting antibodies against additional known autoantigens essentially as described using the pREN2 vector [34]. These new constructs included Ku, Trim-28, ribosomal P0 protein (P0), and against a C-terminal fragment of PM/Scl. DNA sequencing was used to confirm the integrity of all newly described autoantigen constructs. A complete list of the LIPS antigens used along with their characteristics and notation about whether they were tested in a blinded fashion is provided in Table 2.

**Table 2:** Summary of Seropositivity in the Twins and Healthy Controls

**Autoantigen**^**a**^	**Seropositive matched control**	**Seropositive unaffected**	**Seropositive affected**	**Fischer’s exact testing unaffected vs. affected**	**Reference/comment**
	**N = 31**	**N = 31**	**N = 31**		
**Ro52**	0	0	6	*P* = 0.024	[24,28]
**Ro60**	0	0	5	*NS*	[24,28]
**U1-70 K**	0	0	5	*NS*	[28]
**RNP-A**	0	0	5	*NS*	[28]
**La**	0	0	2	NS	[24,28]
**Smith**	1	0	3	NS	[28]
**Jo-1**	0	0	0	NS	[24]
Ku	0	0	2	NS	New
PML	0	0	2	NS	New
P0	0	0	0	NS	New
Trim-28	0	0	0	NS	New
**TPO**	1	1	0	NS	[24,25]
**TGM2**	1	1	0	NS	[28]
**ATP4B**	0	0	5^b^	*NS*	[24,25]
**IL-1α**	1	0	1	NS	[23,35]
**IFN-α**	0	0	1	NS	[23,28,35]
**IFN-γ**	2	1	1	NS	[23,28,35]
**IFN-ω**	0	0	0	NS	[23,28,35]
TNF-β	0	0	1	NS	[23,35]
GMCSF	0	0	0	NS	[23,35]
IL-17	0	0	0	NS	[23,35]
**Total**	**6**^**c**^	**3**^**c**^	**13**^**c**^	***P*** **= 0.0078**	

^a^Antigens tested in blinded fashion are bolded.

^b^All 5 seropositive subjects were from DM affected twins.

^C^Total number of seropositive subjects in each group.

### LIPS testing

LIPS testing was performed as described in a detailed protocol and video [36]. Briefly, for testing of the cohort, a master plate of the serum samples was first constructed in a deep well master plate by diluting serum 1/10 in buffer A (50 mM Tris, pH 7.5, 100 mM NaCl, 5 mM MgCl_2_, 1% Triton X-100 and 0.001% bromophenol red). For LIPS analysis, 40 μl of buffer A, 10 μl of diluted sera from the master plate (1 μl equivalent), and 1 × 10^7^ light units (LU) of *Renilla* luciferase-antigen Cos1 cell extract were added to a final volume of 100 μl) to each well of a standard polypropylene plate. After incubation for 1 hour at room temperature on a rotary shaker, the 100 μl antigen-antibody reaction mixture was transferred to a 96-well filter plate containing 5 μl of a 30% suspension of protein A/G beads and further incubated with shaking. For detecting anti-TGM2 IgA autoantibodies, goat anti-human IgA-agarose conjugated beads (Sigma) were substituted for protein A/G beads. After 60 minutes of incubation, the filter plates containing the bead-immobilized antibody-antigen complexes were washed using a BioMek robotic workstation with a vacuum manifold. The LU of the filter plates were then measured in a Berthold LB 960 Centro microplate luminometer (Berthold Technologies, Bad Wilbad, Germany) using coelenterazine substrate mix (Promega, Madison, WI). Additional positive control sera and testing was also used for validating the diagnostic potential of some of the antigens. All data represent raw antibody levels without subtracting the buffer blanks. Based on known cut-offs for most of the autoantigens, seropositivity status was determined before the codes were broken. For the new autoantigens generated for this study, cut-off values were calculated based on the mean plus 3 standard deviations of the healthy controls.

### Statistical analysis and heatmap analysis

The antibody levels in the cohort were analyzed using the GraphPad Prism software (San Diego, CA). Since this study was exploratory, *P* values were not corrected for multiple comparisons and P values less than 0.05 was deemed as statistically significant. The non-parametric Mann-Whitney *U* statistical test was used for comparison of antibody levels in the different groups. For comparing the seroprevalence in the different groups, contingency tables were generated and analyzed using the Fischer’s exact test for statistical significance.

A heat map was employed for visualization of the spectrum and intensity of autoantibody responses in the individual seropositive twin pairs. For construction of the heatmap, the corresponding twin without autoantibodies was used as a reference group to determine the fold increase compared to the seropositive twin and was color-coded according to the key.

## Results

### Clinical characteristics of the twin cohort

A cohort of 31 disease-discordant twin pairs and 31 matched, healthy controls was utilized to study the prevalence of autoantibodies against a panel of defined human autoantigens. The clinical characteristics of the affected twins are described in Table 1 including the age, gender, autoimmune disease diagnosis, mono- and dizygotic twin status, disease duration and treatment status. Of the 31 twin pairs, 71% were monozygotic and 29% were dizygotic (Table 1). Clinical diagnoses of the affected twins with autoimmune disease were DM (67.7%, 21/31), SLE (29.0%, 9/31) and PM (3.2%, 1/31). The average age of the twins in the cohort was 14.2 ± 2.1 years and most of the affected twins (93.5%) were being treated with immunosuppressive agents at the time of testing. A complete list of the available clinical information in the affected twins with autoimmune disease is provided (Table 1).

### LIPS screening for autoantibodies against the major SLE autoantigen targets

**Figure 1:**
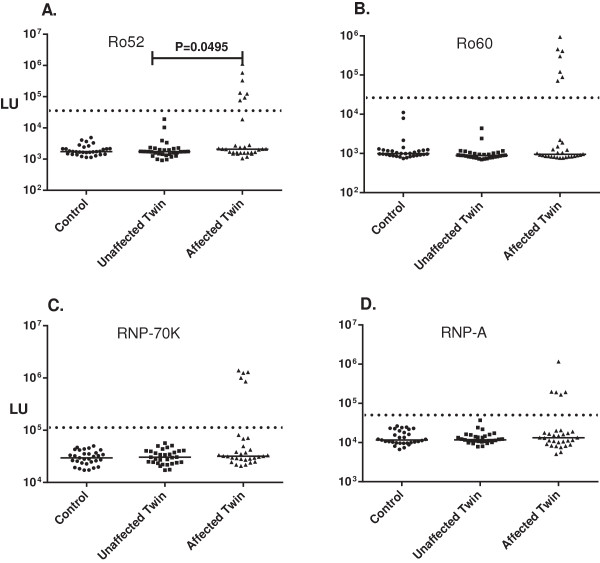
**Autoantibodies against lupus–associated autoantigens in the twin cohort.** Samples from 93 subjects were evaluated for autoantibodies against **(A.)** Ro52, **(B.)** Ro60, **(C.)** RNP-70 k and **(D.)** RNP-A autoantigens. The individual autoantibody levels were plotted on the Y-axis using a log_10_ scale and the median autoantibody value in each group is shown by the horizontal line. Only statistically significant *P* values between the two groups are shown and were calculated using the Mann-Whitney *U* test. A pre-determined cut-off value (stippled line) along with seropositive status was assigned before the identity codes of the blinded samples were broken.

In total, 21 autoantigens were tested by LIPS (Table 2). The rationale for examining antibody responses against many of the autoantigens was their known associations with autoimmune diseases such as T1D, SLE and myositis. Initially, 14 different autoantigens were tested in a blinded fashion. Prior to un-blinding the sample codes, cut-off values for seropositivity were established using previously defined cutoff values. Seven additional autoantigens were tested subsequently (i.e., after the sample codes were broken) in an effort to identify additional informative autoantibody responses. Analysis of autoantibody production to four of the major SLE autoantigens revealed seropositive responses in a subset of affected twins with autoimmune disease. No seropositive responses were detected in either the unaffected twins or healthy blood donors (Figure 1 and Table 2). The most informative autoantibody responses were against Ro52, one of the two major components comprising the SSA antigen, in which Ro52 seropositivity was detected in a total of six affected twins: four with DM and two with SLE (Figure 1A). Autoantibody levels for the Ro52 seropositive subjects showed robust levels that were 10 to 160-fold higher than the unaffected twins and healthy controls. Autoantibodies against Ro60, RNP-A, and RNP- 70 K were also detected in 5, 4 and 4 of the affected twins, respectively, but were not found in any of the unaffected twins or blood donors (Figure 1 and Table 2). As a group, only the elevated Ro52 autoantibody levels in the affected twins compared to unaffected twins (or healthy controls) was statistically significant (Mann Whitney *U* test; *P* = 0.0495).

**Figure 2:**
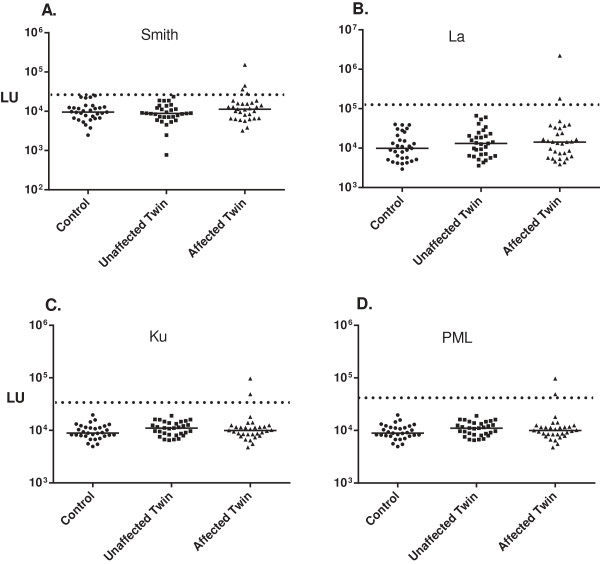
**Autoantibodies against additional autoantigen targets in the twin cohort.** Samples from 93 subjects were evaluated for autoantibodies against **(A.)** Sm, **(B.)** La, **(C.)** Ku and **(D.)** PML antigens. The individual autoantibody levels are plotted on the Y-axis using a log_10_ scale and the median antibody value in each group is shown by the horizontal line. While a pre-determined cut-off along was seropositive status was assigned for La and Smith before the sample ID codes were revealed, the cut-off shown for the newly developed PML and Ku autoantigens were based on differences greater than the mean plus 3 standard deviations.

Some affected twins with autoimmune disease, but none of the unaffected twins or healthy controls, showed autoantibodies against other autoantigens associated with systemic disease including La, PM/Scl and Ku (Figure 2). In the case of the Sm autoantigen, one of the healthy controls (1/31) was seropositive, but it was a low autoantibody titer just above the cut-off value. There were no significant autoantibodies detected above the cut-off values against several other potential diagnostic antigens including Jo-1, PO, or Trim-28 (Table 2). Control assays demonstrating the presence of high titer autoantibody levels against Jo-1 and P0 autoantigens in other myositis and SLE patients, respectively, with pre-established positive seroreactivities supported the validity of these tests (data not shown).

The frequency of autoantibody responses was also calculated for the eight autoantigens (Ro52, Ro60, RNP-70 K, RNP-A, SM, La, Ku and PML) seen in systemic autoimmune diseases that showed seropositivity in the cohort. From this analysis, 32% (7/22) of the twins with myositis and 44% (4/9) of the twins with SLE demonstrated seropositivity (Table 2). Analysis by Fischer’s exact testing showed only the increased frequency of autoantibodies against Ro52 in the affected vs. the unaffected twins was statistically significant (*P* < 0.05). While 11 of the affected twins with SLE or myositis demonstrated seropositivity, none of the healthy twins (0/31) had autoantibodies against these targets. Evaluation by the Fischer’s exact test revealed that only the affected (n = 12) and not the unaffected twins (n = 0) had autoantibodies against the systemic autoantigen targets, and this difference was highly significant (P < 0.0001). Similarly, as expected, the affected twins had significantly higher frequencies of autoantibodies against these systemic autoantigen targets compared to the unrelated, healthy controls (P < 0.0001). Thus, our findings strongly suggest that autoantibodies against these targets are largely found in the affected twins and not in the unaffected twins or healthy unrelated controls.

### Autoantibodies against the gastric ATPase in the affected twins with DM

**Figure 3:**
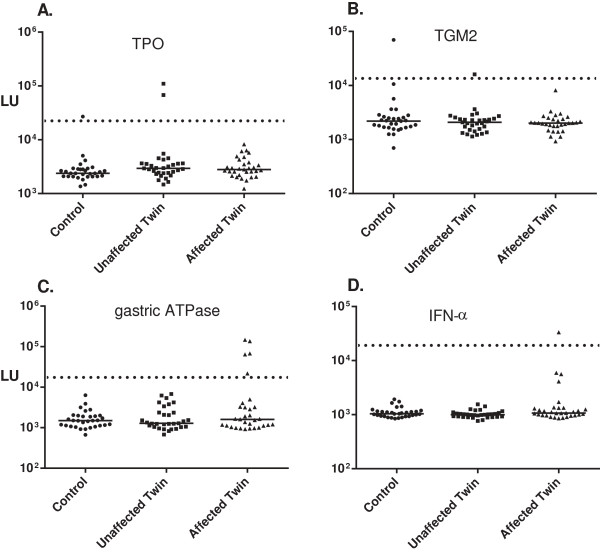
**High prevalence of anti-gastric autoantibodies in the affected twins with myositis.** Samples from 93 subjects were evaluated for autoantibodies against **(A.)** TPO, **(B.)**TGM2, **(C.)** gastric ATPase and **(D.)** IFN-α. The individual autoantibody levels are plotted on the Y-axis using a log_10_ scale and the median antibody value in each group is shown by the horizontal line. Only the statistically significant *P* values between the two groups are shown and were calculated using the Mann-Whitney *U* test. For these four autoantigens, pre-determined cut-off values along with the seropositive status were assigned before the sample codes were revealed.

Autoantibody responses in the cohort were also evaluated against several autoantigens associated with other autoimmune conditions including the TPO autoantigen present in autoimmune thyroid disease, TGM2 associated with colon inflammation in celiac disease and the gastric ATPase associated with autoimmune gastritis [25]. From LIPS analysis, seropositivity against TPO was detected in one healthy control and one unaffected twin (Figure 3A). Similar results were found for TGM2, in which one blood donor and one unaffected twin was seropositive (Figure 3B). Interestingly, high levels of autoantibodies against the gastric ATPase were only found in five of the affected twins, but not in healthy twins or control subjects (Figure 3C). Autoantibody levels for the anti-ATPase seropositive subjects showed robust levels that were 10-160 fold higher than the unaffected twins and healthy controls. Examination of the clinical diagnosis of the five anti-gastric ATPase seropositive subjects revealed that they were all affected twins with DM thereby showing an overall prevalence of 23.8% in DM. Anti-gastric ATPase autoantibodies have not been described before in myositis and further studies are needed to assess its clinical usefulness.

### Paucity of anti-cytokine autoantibodies in the twin cohort

While cytokines have a well-known association with inflammation, anti-viral responses and autoimmunity, less is known about the presence of autoantibodies against cytokines in different autoimmune disease cohorts. Previously, autoantibodies against cytokines have been identified in several autoimmune conditions including autoimmune polyendocrinopathy syndrome type I [37] and SLE [28,38]. From testing the twin cohort for autoantibodies against seven cytokines (IL-1α, IFN-α, IFN-ω, IFN-γ and IL-17, GMCSF and TNF-β), only a few individuals were found to have significant seropositivity. In the case of IFN-α autoantibodies, one healthy control and one SLE patient were found to be seropositive (Figure 3D and Table 2). Additional sporadic seropositivity was detected: a single affected twin with DM had autoantibodies against TNF- β; IL-1α autoantibodies were found in one control subject and one affected twin with DM; and IFN-γ autoantibodies were detected in three healthy controls, a healthy twin, and one affected twin with DM (Table 2). No autoantibodies were detected in any of the subjects against IFN- ω, IL-17 and GMCSF.

### Heatmap analysis and clinical correlates of the seropositive twin pairs

**Figure 4:**
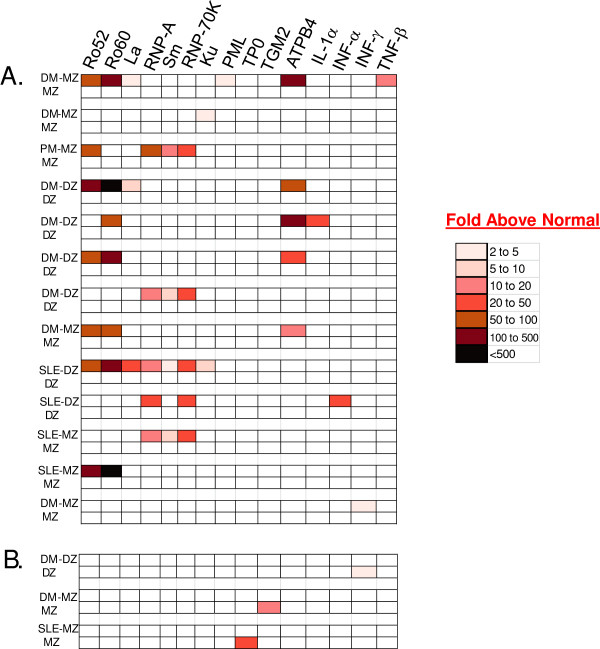
**Heatmap analysis of autoantibody seropositive twins.** Heatmap analysis shows autoantibody seropositivity against the 15 autoantigens that were found in at least one of the twin pairs. Relative immunoreactivity against target autoantigens are shown for the 13 affected twins **(panel A)** and in 3 unaffected twins **(panel B)**. As described in Materials and Methods, each twin pair was color-coded reflecting the relative-fold elevation in autoantibody levels among seropositive twins compared to their seronegative counterparts.

From testing the 21 autoantigen panel, 15 autoantigens showed significant immunoreactivity with at least one of the unaffected or affected twins (Table 2). To further understand individual twin immunoreactivity, a heatmap analysis was used. Since no twin pair shared seropositivity against the same autoantigen, a color code was used to denote the relative-fold elevation in the autoantibody levels in the seropositive twin above the corresponding seronegative twin (Figure 4). As shown in the heatmap, 13 affected twins and 3 unaffected twins showed autoantibody responses against at least one autoantigen in the panel. While eleven of the thirteen affected twins showed autoantibodies against two or more autoantigens, the 3 seropositive, unaffected twins showed autoantibodies only against one autoantigen (Figure 4). In the affected twins, the highest multiple seropositive subjects had DM and SLE showing immunoreactivity against 6 and 7 autoantigens, respectively. Importantly, only the affected twin group had high levels of autoantibodies that were 50 to < 500-fold higher than seronegative twins (Figure 4). Among the monozygotic twins, 31.8% (7/22) were seropositive, while only 4.5% (1/22) of the unaffected, monozygotic twins was seropositive (*P* = 0.046). Additionally, there were a total of 20 seropositive responses in the 7 affected monozygotic twins, but only one autoantibody response was found in one unaffected, monozygotic twin. In myositis, three of the 22 cases were from adults, but only the one subject with polymyositis showed significant autoantibodies. Lastly, inspection of the five ATPase seropositive DM patients revealed that they were all co-positive for Ro60, as well as one additional autoantigen, suggesting that the anti-gastric autoantibodies in myositis reflected existing high levels of auto-reactivity (Figure 4). Overall, these autoantibody profiles highlight the few, single immunoreactivities and low titer autoantibodies found in the unaffected twins compared to the highly robust autoantibody levels against multiple autoantigens seen in the affected twins. The autoantibody profiles were also analyzed in relation to the severity of disease in the subjects. The severity of autoimmune disease as measured by the physician global disease activity was higher in the 13 seropositive subjects (median score = 22.0) compared to the 18 seronegative subjects (median score = 8.5) (*P* = 0.019). Similarly, the global disease damage score was also significantly increased in the autoantibody positive patients (*P* = 0.047).

## Discussion

While autoimmune diseases have high morbidity and mortality, little is known about the cause of most autoimmune disorders [39]. Here autoantibody profiles were used to study a cross-sectional cohort of mono-and dizygotic twins discordant for myositis and SLE. The overall data provides compelling evidence that autoantibodies selectively segregated in the twins with autoimmune disease and were not prevalent in the corresponding unaffected twin or in matched controls. While the presence of autoantibodies may indicate sustained disease activity in the affected twins, the reduced seropositivity in the unaffected twins may indicate a lack of detectable subclinical disease. Additionally, there were no significant differences in autoantibody levels between the unaffected twins and healthy controls. These findings suggest that there are no intermediate autoantibody levels in the unaffected twins between the affected twins and healthy controls.

Although three of the unaffected twins had autoantibody responses, the autoantibody levels were low and directed against three autoantigens, TPO, TGM2 and IFN-γ, which are not typically associated with systemic autoimmunity. Autoantibodies against TPO and TGM2 are also common in the general population, while the low levels of autoantibodies against IFN-γ in one unaffected twin was unlikely to have neutralizing cytokine activity. Moreover, the corresponding affected twins in each case were not found to harbor these three autoantibodies. The presence of autoantibodies in three unaffected twins may reflect normal, acute inflammatory conditions, such as those seen in response to infection, or possibly other immune-mediated abnormalities observed among relatives of patients with autoimmune conditions. A key finding in our study was that 31.8% of the monozygotic affected twins were autoantibody seropositive vs. only 4.5% of the unaffected, monozygotic twins (*P* = 0.046) suggesting that the production of autoantibodies likely involves more than genetic risk factors requiring additional epigenetic or environmental factors for inducing disease [4,5].

Based on our previous study, approximately 90% of SLE patients demonstrated autoantibodies against five of the candidate autoantigens [28]. Surprisingly, we detected only 44% seropositivity for the SLE affected twins (4/9) and 41% seropositivity for the myositis affected twins (9/22) against this larger autoantigen panel. The lower number of seropositive affected twins may have been due to two features of the cohort. First, the subjects used in the present study were younger (i.e. mean age of 14.2 years) compared to adults that were used in the previous study. Children with these autoimmune manifestations may represent different subsets of patients and would have had the disease for a relatively shorter period of time. Secondly, approximately 90% of the affected twins were receiving treatment, which may have attenuated autoantibody responses. Another potential explanation for the observed relative lack of autoantibodies is that our autoantigen panel did not include either anti-DNA or anti-phospholipids antigens for SLE [40] or other DM-specific autoantigens [41]. A more extended autoantigen panel including several of these additional autoantigens may provide further insights into the nature of the disease-discordant twin pair cohort. Our current findings that affected twins with high autoantibody levels had increased disease activity illustrate the possible clinical usefulness of the LIPS assay approach in serial analyses.

One novel finding of our study was the detection of autoantibodies directed against the gastric ATPase in several myositis patients that were also positive for other autoantigens. Previous studies have identified autoantibodies against the gastric ATPase in other autoimmune conditions including autoimmune gastritis [42], Sjögren’s Syndrome [24], and T1D [25,43]. The finding of gastric autoantibodies in some myositis patients is perhaps consistent with the gastrointestinal symptoms experienced in some patients. A variety of organ-specific autoantibodies have recently been described in JDM patients from Brazil including those against TPO and T1D autoantigens [44]. The autoantibodies observed in our cohort against the gastric ATPase may reflect the B-cell immune dysfunction or epitope spreading in more severe cases of myositis. It would be of interest to further examine longitudinal samples from gastric ATPase seropositive myositis patients to determine the temporal relationship between seroconversion and myositis disease onset. Based on recent findings concerning the role of the intestinal microbiome in autoimmune disease [45], further studies are needed to explore the possibility that the gastrointestinal infections might trigger these autoantibodies.

In contrast to the study by Reichlin et al. [19], we found little evidence for the presence of autoantibodies in otherwise healthy unaffected SLE twins. Another study of ANA autoantibodies in SLE and first-degree relatives (FDR) suggested measurable ANA titers in family members [20]. In a related SLE study, only a small percentage of unaffected family members had autoantibodies against SSA (49.57% with SLE vs. 4.9% FDR) and DNA (79.1% with SLE vs. 4.58% FDR) [21]. Studies with adult discordant twins (i.e. average age > 40 years) for rheumatoid arthritis [46] and Hashimoto’s thyroiditis [47] have also found autoantibodies in the unaffected twins. There are some important differences in these published studies compared to our study. First, our cohort consisted of primarily myositis patients and a smaller number of SLE patients. Most importantly, the age of the subjects from our cross-sectional study was considerably younger (i.e. mean age of 14.2 years) than other published studies. If the genetic background is necessary but not sufficient for autoantibody production, the younger twins examined in our study may have had insufficient time to develop higher titer autoantibodies. Moreover, our study used a highly quantitative assay that employed a panel of autoantigens that focused on specific protein targets. In this study, we did not examine ANA or anti-DNA autoantibodies, which have been characterized more extensively in other studies. It is possible that more subtle clinical findings like ANA or altered proteomic and gene expression profiles are present in genetically-related individuals with and without autoimmune disease [29,30]. For example, intermediate gene expression profiles have been observed in unaffected twins compared with affected twins and healthy controls [30]. Lastly, it is important to point out that our study has several limitations, including the small sample size and combination of adults and children, resulting from the challenges of identifying and recruiting qualified twins discordant for IIM and SLE. Additional studies in larger cohorts will be needed to more completely assess our findings.

## Conclusions

In our cohort of relatively young twins discordant for myositis or SLE, our results show dramatic differences in autoantibody profiles between affected and unaffected twins. The clear difference between the autoantibody levels of the seropositive patients and the subset of corresponding unaffected twins supports the idea that the developmental onset of autoantibody production is controlled by more than genetic risk factors. Based on the observed induction of autoantibodies before disease onset [18], more detailed studies using longitudinal samples from prospective cohorts may allow potential environmental triggers to be examined further by concurrent exposure assays and autoantibody profiling.

## Abbreviations

ANA: Anti-nuclear antibodies; DM: Dermatomyositis; FDR: First-degree relatives; IFN: Interferon; IIM: Idiopathic inflammatory myopathies; LIPS: Luciferase Immunoprecipitation Systems; PM: Polymyositis; SLE: Systemic Lupus Erythematosus; Sm: Smith autoantigen; TGM2: Transglutaminase 2; TPO: Thyroid peroxidase; PO: Ribosomal protein PO.

## Competing interests

The authors declare that they have no competing interests.

## Authors’ contributions

Contribution: LG contributed to study design, sample preparation, data analyses, and manuscript preparation. TPO’H contributed to study design, sample preparation, and manuscript editing. AG contributed to LIPS test, data analysis and manuscript editing. LGR contributed to patient recruitment, clinical assessments, and manuscript editing. FWM contributed to study design, patient recruitment, clinical assessments, and manuscript editing. P.D.B contributed to study design, LIPS test, data analyses, and manuscript drafting and editing. All authors read and approved the final manuscript.

## Pre-publication history

The pre-publication history for this paper can be accessed here:

http://www.biomedcentral.com/1471-2474/15/67/prepub

## References

[B1] Bogdanos DP Smyk DS Rigopoulou EI Mytilinaiou MG Heneghan MA Selmi C Gershwin ME Twin studies in autoimmune disease: genetics, gender and environment J Autoimmun 2012 38 2-3 J156 169 10.1016/j.jaut.2011.11.003 22177232

[B2] Rai E Wakeland EK Genetic predisposition to autoimmunity–what have we learned? Semin Immunol 2011 23 2 67 83 10.1016/j.smim.2011.01.015 21288738

[B3] Bach JF Infections and autoimmune diseases J Autoimmun 2005 25 Suppl 74 80 16278064 10.1016/j.jaut.2005.09.024

[B4] Javierre BM Hernando H Ballestar E Environmental triggers and epigenetic deregulation in autoimmune disease Discov Med 2011 12 67 535 545 22204770

[B5] Miller FW Environmental agents and autoimmune diseases Adv Exp Med Biol 2011 711 61 81 10.1007/978-1-4419-8216-2_6 21627043

[B6] Christen U von Herrath MG Infections and autoimmunity–good or bad? J of Immunol 2005 174 12 7481 7486 15944245 10.4049/jimmunol.174.12.7481

[B7] Delgado-Vega AM Alarcon-Riquelme ME Kozyrev SV Genetic associations in type I interferon related pathways with autoimmunity Arthritis Res Ther 2010 12 1 S2 10.1186/ar2900 20392289 PMC2991775

[B8] Bilgic H Ytterberg SR Amin S McNallan KT Wilson JC Koeuth T Ellingson S Newman B Bauer JW Peterson EJ Baechler EC Reed AM Interleukin-6 and type I interferon-regulated genes and chemokines mark disease activity in dermatomyositis Arthritis and Rheumatol 2009 60 11 3436 3446 10.1002/art.24936 19877033

[B9] Greenberg SA Pinkus JL Pinkus GS Burleson T Sanoudou D Tawil R Barohn RJ Saperstein DS Briemberg HR Ericsson M Park P Amato AA Interferon-alpha/beta-mediated innate immune mechanisms in dermatomyositis Annals of Neurol 2005 57 5 664 678 10.1002/ana.20464 15852401

[B10] Tezak Z Hoffman EP Lutz JL Fedczyna TO Stephan D Bremer EG Krasnoselska-Riz I Kumar A Pachman LM Gene expression profiling in DQA1*0501+ children with untreated dermatomyositis: a novel model of pathogenesis J of Immunol 2002 168 8 4154 4163 11937576 10.4049/jimmunol.168.8.4154

[B11] Baechler EC Batliwalla FM Karypis G Gaffney PM Ortmann WA Espe KJ Shark KB Grande WJ Hughes KM Kapur V Gregersen PK Behrens TW Interferon-inducible gene expression signature in peripheral blood cells of patients with severe lupus Proc Natl Acad Sci USA 2003 100 5 2610 2615 10.1073/pnas.0337679100 12604793 PMC151388

[B12] von Wussow P Jakschies D Hartung K Deicher H Presence of interferon and anti-interferon in patients with systemic lupus erythematosus Rheumatol Int 1988 8 5 225 230 10.1007/BF00269199 3266358

[B13] Blomberg S Eloranta ML Cederblad B Nordlin K Alm GV Ronnblom L Presence of cutaneous interferon-alpha producing cells in patients with systemic lupus erythematosus Lupus 2001 10 7 484 490 10.1191/096120301678416042 11480846

[B14] Bengtsson AA Sturfelt G Truedsson L Blomberg J Alm G Vallin H Ronnblom L Activation of type I interferon system in systemic lupus erythematosus correlates with disease activity but not with antiretroviral antibodies Lupus 2000 9 9 664 671 10.1191/096120300674499064 11199920

[B15] Notkins AL New predictors of disease. Molecules called predictive autoantibodies appear in the blood years before people show symptoms of various disorders. Tests that detected these molecules could warn of the need to take preventive action Sci Am 2007 296 3 72 79 10.1038/scientificamerican0307-72 17348162

[B16] Petri M Review of classification criteria for systemic lupus erythematosus Rheumatic Dis Clin of North Am 2005 31 2 245 254 vi 10.1016/j.rdc.2005.01.009 15922144

[B17] Gunawardena H Betteridge ZE McHugh NJ Myositis-specific autoantibodies: their clinical and pathogenic significance in disease expression Rheumatology (Oxford) 2009 48 6 607 612 10.1093/rheumatology/kep078 19439503

[B18] Arbuckle MR McClain MT Rubertone MV Scofield RH Dennis GJ James JA Harley JB Development of autoantibodies before the clinical onset of systemic lupus erythematosus N Engl J Med 2003 349 16 1526 1533 10.1056/NEJMoa021933 14561795

[B19] Reichlin M Harley JB Lockshin MD Serologic studies of monozygotic twins with systemic lupus erythematosus Arthritis and Rheumatol 1992 35 4 457 464 10.1002/art.1780350416 1567495

[B20] Laustrup H Heegaard NH Voss A Green A Lillevang ST Junker P Autoantibodies and self-reported health complaints in relatives of systemic lupus erythematosus patients: a community based approach Lupus 2004 13 10 792 799 10.1191/0961203304lu2015oa 15540512

[B21] Navarra SV Ishimori MI Uy EA Hamijoyo L Sama J James JA Holers VM Weisman MH Studies of Filipino patients with systemic lupus erythematosus: autoantibody profile of first-degree relatives Lupus 2011 20 5 537 543 10.1177/0961203310385164 21183559

[B22] Burbelo PD Ching KH Bren KE Iadarola MJ Searching for biomarkers: humoral response profiling with luciferase immunoprecipitation systems Expert Rev Proteomics 2011 8 3 309 316 10.1586/epr.11.23 21679112 PMC3818131

[B23] Browne SK Burbelo PD Chetchotisakd P Suputtamongkol Y Kiertiburanakul S Shaw PA Kirk JL Jutivorakool K Zaman R Ding L Hsu AP Patel SY Olivier KN Lulitanond V Mootsikapun P Anunnatsiri S Angkasekwinai N Sathapatayavongs B Hsueh PR Shieh CC Brown MR Thongnoppakhun W Claypool R Sampaio EP Thepthai C Waywa D Dacombe C Reizes Y Zelazny AM Saleeb P Adult-onset immunodeficiency in Thailand and Taiwan N Engl J Med 2012 367 8 725 734 10.1056/NEJMoa1111160 22913682 PMC4190026

[B24] Burbelo PD Leahy HP Issa AT Groot S Baraniuk JN Nikolov NP Illei GG Iadarola MJ Sensitive and robust luminescent profiling of anti-La and other autoantibodies in Sjogren's syndrome Autoimmunity 2009 42 6 515 524 10.1080/08916930902911738 19657778 PMC3417760

[B25] Burbelo PD Lebovitz EE Bren KE Bayat A Paviol S Wenzlau JM Barriga KJ Rewers M Harlan DM Iadarola MJ Extrapancreatic autoantibody profiles in type I diabetes PloS one 2012 7 9 e45216 10.1371/journal.pone.0045216 23028856 PMC3448600

[B26] Burbelo PD Groot S Dalakas MC Iadarola MJ High definition profiling of autoantibodies to glutamic acid decarboxylases GAD65/GAD67 in stiff-person syndrome Biochem and Biophys Res Commun 2008 366 1 1 7 10.1016/j.bbrc.2007.11.077 18047830 PMC2215321

[B27] Ching KH Burbelo PD Kimball RM Clawson LL Corse AM Iadarola MJ Recombinant expression of the AChR-alpha1 subunit for the detection of conformation-dependent epitopes in Myasthenia Gravis Neuromuscul Dis: NMD 2011 21 3 204 213 10.1016/j.nmd.2010.12.003 PMC3046303 21195619

[B28] Ching KH Burbelo PD Tipton C Wei C Petri M Sanz I Iadarola MJ Two major autoantibody clusters in systemic lupus erythematosus PloS one 2012 7 2 e32001 10.1371/journal.pone.0032001 22363785 PMC3283706

[B29] O'Hanlon TP Li Z Gan L Gourley MF Rider LG Miller FW Plasma proteomic profiles from disease-discordant monozygotic twins suggest that molecular pathways are shared in multiple systemic autoimmune diseases Arthritis Res Ther 2011 13 6 R181 10.1186/ar3506 22044644 PMC3315681

[B30] O'Hanlon TP Rider LG Gan L Fannin R Paules RS Umbach DM Weinberg CR Shah RR Mav D Gourley MF Miller FW Gene expression profiles from discordant monozygotic twins suggest that molecular pathways are shared among multiple systemic autoimmune diseases Arthritis Res Ther 2011 13 2 R69 10.1186/ar3330 21521520 PMC3132064

[B31] Bohan A Peter JB Polymyositis and dermatomyositis (second of two parts) N Engl J Med 1975 292 8 403 407 10.1056/NEJM197502202920807 1089199

[B32] Bohan A Peter JB Polymyositis and dermatomyositis (first of two parts) N Engl J Med 1975 292 7 344 347 10.1056/NEJM197502132920706 1090839

[B33] Rider LG Werth VP Huber AM Alexanderson H Rao AP Ruperto N Herbelin L Barohn R Isenberg D Miller FW Measures of adult and juvenile dermatomyositis, polymyositis, and inclusion body myositis: Physician and Patient/Parent Global Activity, Manual Muscle Testing (MMT), Health Assessment Questionnaire (HAQ)/Childhood Health Assessment Questionnaire (C-HAQ), Childhood Myositis Assessment Scale (CMAS), Myositis Disease Activity Assessment Tool (MDAAT), Disease Activity Score (DAS), Short Form 36 (SF-36), Child Health Questionnaire (CHQ), physician global damage, Myositis Damage Index (MDI), Quantitative Muscle Testing (QMT), Myositis Functional Index-2 (FI-2), Myositis Activities Profile (MAP), Inclusion Body Myositis Functional Rating Scale (IBMFRS), Cutaneous Dermatomyositis Disease Area and Severity Index (CDASI), Cutaneous Assessment Tool (CAT), Dermatomyositis Skin Severity Index (DSSI), Skindex, and Dermatology Life Quality Index (DLQI) Arthritis care & Res 2011 63 Suppl 11 S118 S157 10.1002/acr.20532 PMC3748930 22588740

[B34] Burbelo PD Goldman R Mattson TL A simplified immunoprecipitation method for quantitatively measuring antibody responses in clinical sera samples by using mammalian-produced Renilla luciferase-antigen fusion proteins BMC Biotechnol 2005 5 22 10.1186/1472-6750-5-22 16109166 PMC1208859

[B35] Burbelo PD Browne SK Sampaio EP Giaccone G Zaman R Kristosturyan E Rajan A Ding L Ching KH Berman A Oliveira JB Hsu AP Klimavicz CM Iadarola MJ Holland SM Anti-cytokine autoantibodies are associated with opportunistic infection in patients with thymic neoplasia Blood 2010 116 23 4848 4858 10.1182/blood-2010-05-286161 20716769 PMC3321746

[B36] Burbelo PD Ching KH Klimavicz CM Iadarola MJ Antibody profiling by Luciferase Immunoprecipitation Systems (LIPS) J Vis Exp 2009 32 10.3791/1549 PMC3164068 19812534

[B37] Meager A Visvalingam K Peterson P Moll K Murumagi A Krohn K Eskelin P Perheentupa J Husebye E Kadota Y Willcox N Anti-interferon autoantibodies in autoimmune polyendocrinopathy syndrome type 1 PLoS Med 2006 3 7 e289 10.1371/journal.pmed.0030289 16784312 PMC1475653

[B38] Morimoto AM Flesher DT Yang J Wolslegel K Wang X Brady A Abbas AR Quarmby V Wakshull E Richardson B Townsend MJ Behrens TW Association of endogenous anti-interferon-alpha autoantibodies with decreased interferon-pathway and disease activity in patients with systemic lupus erythematosus Arthritis and rheumatism 2011 63 8 2407 2415 10.1002/art.30399 21506093 PMC4028124

[B39] Moroni L Bianchi I Lleo A Geoepidemiology, gender and autoimmune disease Autoimmun Rev 2012 11 6–7 A386 A392 22142547 10.1016/j.autrev.2011.11.012

[B40] Agmon-Levin N Mosca M Petri M Shoenfeld Y Systemic lupus erythematosus one disease or many? Autoimmun Rev 2012 11 8 593 595 10.1016/j.autrev.2011.10.020 22041578

[B41] Casciola-Rosen L Mammen AL Myositis autoantibodies Curr Opin Rheumatol 2012 24 6 602 608 10.1097/BOR.0b013e328358bd85 22955022 PMC3874450

[B42] Toh BH Gleeson PA Simpson RJ Moritz RL Callaghan JM Goldkorn I Jones CM Martinelli TM Mu FT Humphris DC Pettitt JM Mori Y Masuda T Sobieszczuk P Weinstock J Mantamadiotis T Baldwin GS The 60- to 90-kDa parietal cell autoantigen associated with autoimmune gastritis is a beta subunit of the gastric H+/K(+)-ATPase (proton pump) Proc Natl Acad Sci USA 1990 87 16 6418 6422 10.1073/pnas.87.16.6418 1974721 PMC54545

[B43] De Block CE De Leeuw IH Van Gaal LF High prevalence of manifestations of gastric autoimmunity in parietal cell antibody-positive type 1 (insulin-dependent) diabetic patients. The Belgian Diabetes Registry J Clin Endocrinol Metab 1999 84 11 4062 4067 10566650 10.1210/jcem.84.11.6095

[B44] Aikawa NE Jesus AA Liphaus BL Silva CA Carneiro-Sampaio M Viana VS Sallum AM Organ-specific autoantibodies and autoimmune diseases in juvenile systemic lupus erythematosus and juvenile dermatomyositis patients Clin Exp Rheumatol 2012 30 1 126 131 22261392

[B45] Markle JG Frank DN Mortin-Toth S Robertson CE Feazel LM Rolle-Kampczyk U von Bergen M McCoy KD Macpherson AJ Danska JS Sex differences in the gut microbiome drive hormone-dependent regulation of autoimmunity Science 2013 339 6123 1084 1088 10.1126/science.1233521 23328391

[B46] Svendsen AJ Hjelmborg JV Kyvik KO Houen G Nielsen C Skytthe A Junker P The impact of genes on the occurrence of autoantibodies in rheumatoid arthritis. A study on disease discordant twin pairs J Autoimmun 2013 41 120 125 23290888 10.1016/j.jaut.2012.12.001

[B47] Brix TH Hegedus L Gardas A Banga JP Nielsen CH Monozygotic twin pairs discordant for Hashimoto's thyroiditis share a high proportion of thyroid peroxidase autoantibodies to the immunodominant region A. Further evidence for genetic transmission of epitopic "fingerprints" Autoimmunity 2011 44 3 188 194 10.3109/08916934.2010.518575 20883148

